# Explainable Ensemble Learning and Multilayer Perceptron Modeling for Compressive Strength Prediction of Ultra-High-Performance Concrete

**DOI:** 10.3390/biomimetics9090544

**Published:** 2024-09-09

**Authors:** Yaren Aydın, Celal Cakiroglu, Gebrail Bekdaş, Zong Woo Geem

**Affiliations:** 1 Department of Civil Engineering, Istanbul University-Cerrahpaşa, 34320 Istanbul, Turkey; yaren.aydin1@ogr.iuc.edu.tr; 2 Department of Civil Engineering, Turkish-German University, 34820 Istanbul, Turkey; cakirogl@ualberta.ca; 3 Department of Smart City, Gachon University, Seongnam 13120, Republic of Korea

**Keywords:** UHPC, SHAP, compressive strength, stacking regressor, XGBoost, ANN

## Abstract

The performance of ultra-high-performance concrete (UHPC) allows for the design and creation of thinner elements with superior overall durability. The compressive strength of UHPC is a value that can be reached after a certain period of time through a series of tests and cures. However, this value can be estimated by machine-learning methods. In this study, multilayer perceptron (MLP) and Stacking Regressor, an ensemble machine-learning models, is used to predict the compressive strength of high-performance concrete. Then, the ML model’s performance is explained with a feature importance analysis and Shapley additive explanations (SHAPs), and the developed models are interpreted. The effect of using different random splits for the training and test sets has been investigated. It was observed that the stacking regressor, which combined the outputs of Extreme Gradient Boosting (XGBoost), Category Boosting (CatBoost), Light Gradient Boosting Machine (LightGBM), and Extra Trees regressors using random forest as the final estimator, performed significantly better than the MLP regressor. It was shown that the compressive strength was predicted by the stacking regressor with an average R^2^ score of 0.971 on the test set. On the other hand, the average R^2^ score of the MLP model was 0.909. The results of the SHAP analysis showed that the age of concrete and the amounts of silica fume, fiber, superplasticizer, cement, aggregate, and water have the greatest impact on the model predictions.

## 1. Introduction

In the developing world, the need for high performance concrete is increasing day by day. As a result of both this need and the development of technology, the strength required from concrete is increasing. High-strength concrete is important in reducing the size of structural elements and therefore the total weight of the structure. The construction of lightweight structures also saves on low transportation costs, labor fees, and maintenance fees.

Since resources in the world are limited, it is important to use the available resources sustainably. For example, in the transportation sector, every 100 kg of weight reduced from the car saves 0.35 L of fuel per 100 km in fuel consumption. Thanks to this fuel saving, there is a 9 g reduction in CO_2_ emitted per kilometer [1]. In the field of health, prosthetic feet are also used, which facilitate shock absorption and weight transfer to the front thanks to their light and flexible structure [2]. Sustainability can also be achieved by using lightweight elements in the building sector. One of the materials that can be used for this is ultra-high-performance concrete (UHPC).

UHPC is a new type of cementitious composite with high compressive strength (more than 150 MPa) and superior durability. Its compressive strength ranges from 200 MPa to 800 MPa. Ultra-high-performance concrete is obtained by achieving good adhesion at the aggregate-matrix interface and the densest possible matrix. A cylinder with a compressive strength of 155 MPa can be produced with self-compacting UYPB without hot curing or any other special treatment [3].

The aim of producing UHPC is to develop a single material that is stronger than normal concrete and has many properties, including pore voids, microcracks, and superior load-bearing capacity [4]. One of the production aims of UHPC is to provide earthquake resistance by providing greater deflection and higher energy absorption with lighter elements and reduced cross-sectional areas [3]. This contributes to the design of light but strong structures. Building thin and durable structures using UHPC helps to increase sustainability. By using lightweight concrete as a building material, the building load is reduced. Thus, benefits such as economic and earthquake resistance can be achieved.

**Figure 1:**
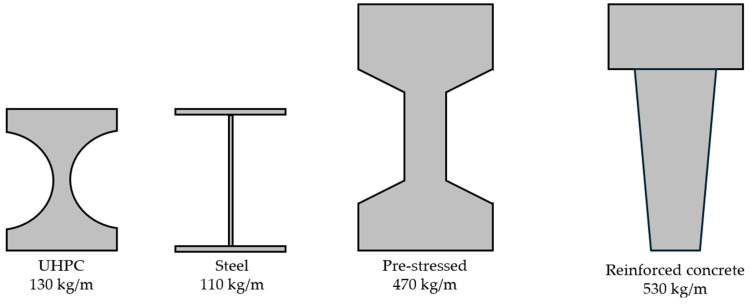
Beams with equal load-carrying capacity [6].

UHPC usually contains more cement than the amount used in normal concrete. Considering both the cost of cement and its environmental impact, it is a more expensive material with a higher CO_2_ footprint. However, since the elements can be designed thinner, the actual volume of concrete used is greatly reduced, and the economic and environmental impacts are also reduced. Figure 1 shows a comparison of the load carrying capacity and the volume of material required to achieve similar mechanical properties compared to other concrete [5].

UHPC is resistant to corrosion and have high durability, which extends the design life of the project and reduces maintenance cost [7]. UHPC has many different uses. One of them is the storage of nuclear waste due to their very superior microstructure properties [8]. The mechanical properties of UHPC are given in Table 1 [3].

**Figure 2:**
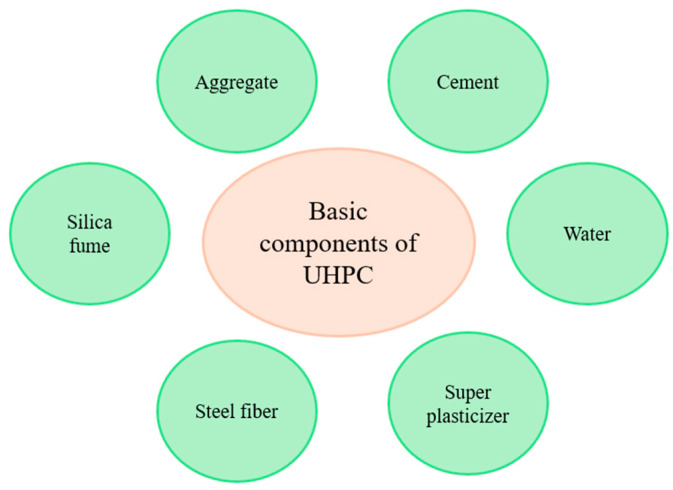
Basic components of UHPC [3].

The strength of high-strength concrete depends on the void structure of the cement paste, the properties of the aggregate, and the properties of the aggregate–cement paste transition zone. The properties of the cement paste and the interface transition zone can be improved by reducing the water/cement ratio and by reducing the maximum grain diameter of the aggregate [9]. Figure 2 shows the basic components of UHPC.

**Figure 3:**
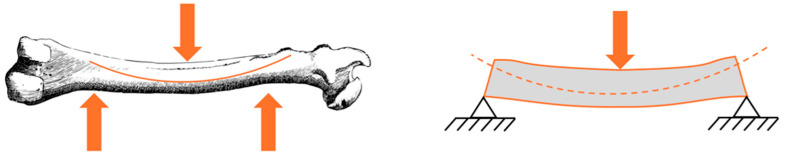
Forces after compression for bone and concrete.

**Figure 4:**
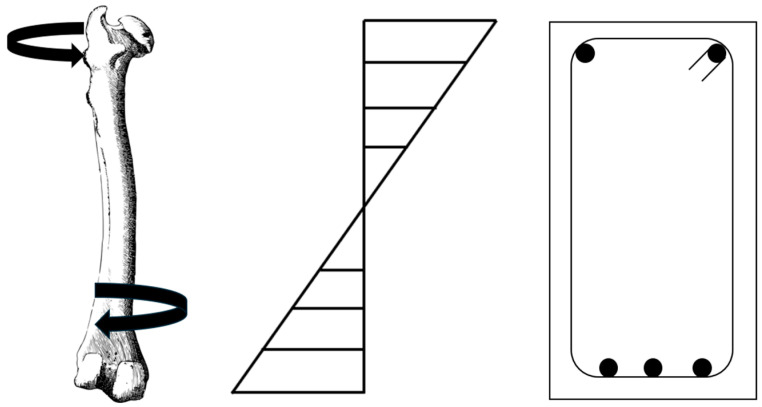
Similarity of bone and concrete.

Concrete and bone have similar properties. Biomechanical tests are used to learn how bone can fracture, what kind of structural changes in bone facilitate fracture in various pathological conditions, and which treatments should be applied and how [10]. When the geometry of bone is evaluated at the organ level, terms such as force, deformation, stiffness, work to failure, and when evaluated at the tissue level, terms such as strength, stress, strain, elasticity modulus, toughness, are used [11]. In biomechanical studies, loading types can be compressive, tensile, bending, torsion, or multiaxial along the long axis of the bone [12]. The same terms apply to concrete. Concrete also has mechanical properties such as compression, tension, and impact response. Whether the system is a skeleton or a structure, it exhibits similar behavior when exposed to similar loads. Only the material used and the incoming load grades are different. Figure 3 shows the compressive and tensile forces after compression in bone and the compressive forces after compression in concrete. This shows the working similarity of bone and concrete. The stress distributions in the concrete compression zone at the moment when the bearing capacity is reached in bone and concrete are similar to each other (Figure 4).

Until this part of the paper, UHPC and bionic similarities have been mentioned. The rest of the article will focus on the prediction of UHPC compressive strength using machine learning (ML). There are many studies on the prediction of the compressive strength of UHPC, which is the subject of this study. Yuvaraj et al. [13] utilized relevance vector machine-based regression to predict the fracture properties and damage load (Pmax) of high-strength and ultra-high-strength concrete beams with MATLAB. As a result, the predicted and actual values obtained by applying the vector machine models were found to be consistent with each other. Marani et al. [14] used random forest, extra trees and gradient-boosting regression to predict the compressive strength of UHPC. As a result, the models used showed high prediction success. Solhmirzaei et al. [15] used different machine-learning techniques such as support vector machine (SVM) and artificial neural networks (ANNs) to predict the failure mode and shear capacity of UHPC beams. The study demonstrated the effectiveness of data-driven ML models for predicting the failure mode and shear capacity of UHPC beams. Jiang et al. [16] used genetic algorithm (GA) and support vector regression (SVR) together to predict the shear strength capacity of medium and ultra-high-strength concrete beams. This hybrid model showed high performance in shear strength prediction with a coefficient of determination (R^2^) of 0.9642 and low error values. Kumar et al. [17] used Gaussian Progress Regression (GPR), Support Vector Machine Regression (SVMR), Ensemble Learning (EL), and optimized versions of these techniques to predict the compressive strength of Lightweight Concrete (LWC). As a result, the optimized GPR showed the highest prediction success with R = 0.9803. Shen et al. [18] used Extreme Gradient Boosting (XGBoost), Adaptive Boosting (AdaBoost), and Bagging to predict the compressive strength of UHPC. Among these techniques, XGBoost performed the best, with a higher R^2^ value (0.90) and lower errors. Liu [19] proposed a new approach based on different meta-heuristic algorithms including the Dynamic Arithmetic Optimization Algorithm (DAOA) to improve the accuracy when predicting UHPC compressive strength (CS) using SVR. SVDA showed the highest performance in this approach. Hiew et al. [20] used three deep feed-forward neural network models to predict the ultimate stress and stress–strain behavior of UHPC. Their proposed ANN exhibited robust prediction performance. Zhu et al. [21] developed a new prediction model using ANN and SVM to predict the 7-day compressive strength of UHPC. The predicted values obtained by ANN were very close to the actual values. Nguyen et al. [22] predicted the compressive strength of UHPC using XGBoost models showed the highest prediction success. Gong and Zhang [23] predicted the compressive strength of UHPC using hybrid machine-learning models, including Adaptive Network Fuzzy Inference System (ANFIS) and metaheuristic algorithms. The results showed that the model using ANFIS and Honey Badger Algorithm together achieved the highest accuracy. Ye et al. [24] used machine learning (ML) to predict the shear strength of ultra-high-performance concrete (UHPC) beams. When the models were compared using performance metrics, the XGBoost and Category Boosting (CatBoost) models were the most successful in prediction. Zhang et al. [25] used SVR in combination with grasshopper optimization algorithm (GOA) and arithmetic optimization algorithm (AOA) to calculate the compressive strength of UHPC. AOA–SVR showed the highest performance, with an R^2^ = 0.917. Nguyen et al. [26] predicted the compressive strength of UHPC with mixtures as input variables. Among the models used, automatic feature engineering and selection (Autofeat) provided the highest success. Li et al. [27] used machine-learning (ML) models, random forest (RF), support vector machine (SVM), and k-nearest neighbor (KNN) to predict the compressive strength of ultra-high-performance concrete. They also used particle swarm optimization (PSO), insect antenna search (BAS), and snake optimization (SO) algorithms inspired by the behavior of living things to optimize the hyperparameters of these ML models. As a result, SO–RF showed the highest prediction success.

There has been several recent research on explainable machine-learning models and their applications in material science. For example, Wakjira and Alam [28] performed the performance-based seismic design of UHPC bridge columns using an explainable machine-learning model. Zhu et al. [29] investigated the creep behavior of UHPC using ML and SHAP. Das and Kashem [30] predicted the compressive and flexural strengths of UHPC with a hybrid ML model using SHAP.

With the production of lightweight structures, both economical and environmentally friendly designs can be made. Predicting the pressure response of UHPC, which is one of the materials that can be used for this, will provide great convenience in construction works. This shows the significance of this study. Multilayer perceptron (MLP) and Stacking Regressor were used in this study. MLP is a neural network consisting of an input layer, one or more hidden layers, and an output layer. Stacking Regressor is a model that combines basic models using a meta-model. These methods are described in detail in Section 2.

In this study, an introduction to the topic and studies using ML in the literature are given in Section 1. The ML models used in this study, the introduction of the dataset and the performance evaluation metrics used are given in Section 2. Section 3 contains the results obtained for the prediction of the compressive strength of UHPC. Section 4 discusses the findings, and, finally, conclusions and recommendations are given in Section 5.

## 2. Materials and Methods

### 2.1. Multilayer Perceptron (MLP)

Artificial neural networks (ANNs) were first introduced to the literature in 1943 by neurophysiologist Warren McCulloch and mathematician Walter Pitts [31]. Artificial neural networks are at the center of the concept of deep learning. They are versatile, powerful, and scalable, making them ideal for tackling large and complex machine-learning tasks, such as classifying billions of images [32]. Deep-learning methods include feed-forward neural networks or multilayer perceptrons with many hidden layers [33].

Multilayer perceptrons consist of an input layer, one or more layers called hidden layers and a final layer called the output layer. In a Multilayer Sensor, an output value is obtained at the end of the output layer. Between the output value and the target value, a loss value is calculated using the loss function and the backpropagation algorithm starts using optimization algorithms. With the backpropagation algorithm, weights and deviation values are updated. The forward propagation and backpropagation algorithms are performed in a loop. In this way, the optimal weights and bias values are calculated to minimize the loss value at the output layer [32].

**Figure 5:**
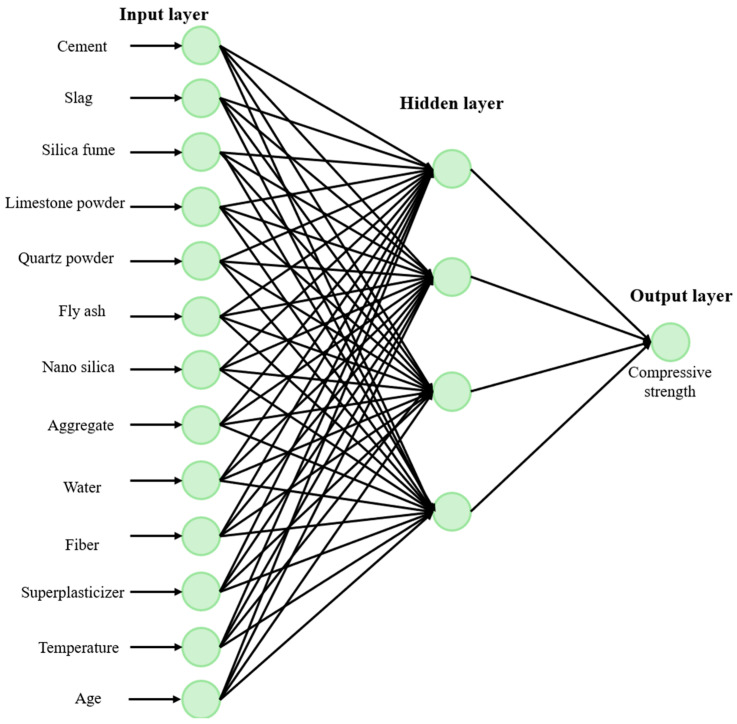
Multilayer perceptron.

In multilayer perceptrons, each perceptron is connected to the perceptron in the next layer [34]. The number of perceptrons in the input layer is the same as the number of features in the dataset. The number of hidden layers can be one or more than one. The number of perceptrons in each hidden layer and the number of perceptrons in the output layer vary according to the problem [35]. Figure 5 shows the multilayer perceptron.

Since there are 13 perceptrons in the input layer in Figure 5, it is understood that there are 13 features in the dataset. There is 1 hidden layer, and the hidden layer consists of 4 sensors. Since there is one perceptron in the output layer, it is understood that there is one output.

In this study, Scikit–Learn library for multilayer perceptron is used. The MLP model consists of an input layer, an output layer, and 3 hidden layers. The number of neurons in the hidden layers are 8, 8, and 2, respectively. The number of hidden layers and the number of neurons in the hidden layers were found with the help of the Grid Search algorithm. The ReLU activation function is used in this study. Adam Optimization was used as the optimization algorithm. The random initial value of the weights was 1. The learning rate was set to 0.1. Default values of other hyperparameters were used.

### 2.2. Stacking

**Figure 6:**
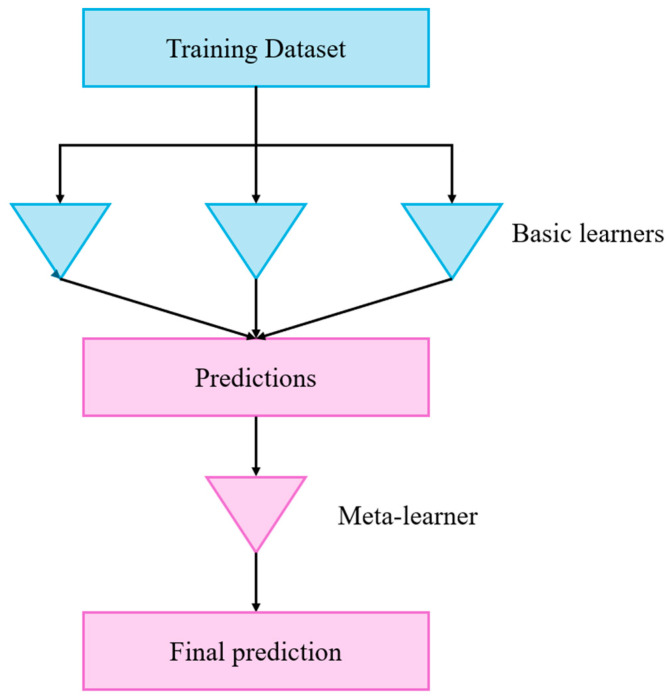
Stacking [38].

The Stacking ensemble regression method proposed by Wolpert [36] basically consists of two stages. In the first stage, the predictions of the methods used in ensemble regressors are obtained and in the next stage, these predictions are processed by a meta-regressor to produce the final prediction of the ensemble. This second part is called the meta-learner, where a new prediction is extracted from the predictions, as shown in Figure 6. Thus, it is aimed to increase the accuracy of the inaccurate predictions that may arise from a single regressor by using ensemble regressors with multiple regressor predictions [36]. Trial and error is used to decide which models will perform better. Stacking is applied to models generated by different learning algorithms [37].

The learning models used for stacking in this study are XGBoost, CatBoost, Light Gradient Boosting Machine (LightGBM), and Extra Trees regressors. Between Section 2.3 and Section 2.6, there are descriptions of these models.

### 2.3. Extreme Gradient Boosting (XGBoost)

XGBoost is an optimized and scalable machine-learning model based on a decision tree and gradient-boosting algorithm. It is so named because it resembles a well-established tree with many leaves. It is designed to provide high performance and superior results in machine-learning tasks. It is an ensemble learning method that combines the predictions of multiple weak models to produce a stronger prediction [39]. Normalization of the objective function is used to reduce model complexity, avoid overfitting, and make the learning process faster [40].

### 2.4. Category Boosting (CatBoost)

The CatBoost model, developed with gradient boosting, is a machine-learning method that achieves high performance by quantifying categorical features. Its name comes from the combination of “Category” and “Boosting”. The algorithm has advantages such as high learning speed, the ability to work with both categorical and numerical data, and visualization options. It differs from traditional gradient-based decision tree algorithms in that it considers categorical attributes during the training period instead of the preprocessing period [41].

### 2.5. Light Gradient Boosting Machine (LightGBM)

The LightGBM algorithm is a gradient-based decision tree used to solve classification and regression problems. It has advantages such as a high processing speed and can be used on big data. In this algorithm, instead of finding separation points in continuous feature values, a histogram-based algorithm divides the continuous feature values into discrete bins and generates feature histograms using these bins during training. In this method, the continuous variables in the training set are converted into discrete variables, which reduces the time and memory usage cost of the model [42].

### 2.6. Extra Trees

Extra Trees is a tree-based algorithm. In the Extra Trees method, copies of the dataset are used to train the model and the branching of nodes is random. The reason for this is to reduce the complexity and computational burden in solving data analysis problems. In this algorithm, the predictions of all individual decision trees are averaged. The predictions of the trees are aggregated to give the final prediction result by arithmetic mean in classification regression problems [43].

### 2.7. Dataset Description

The source of the dataset used in the study is the Data UHPC [44] dataset from the literature. This dataset used for the prediction of the compressive strength of UHPC consists of 890 rows. It has a total of 13 inputs and 1 output. The inputs are cement, slag, limestone powder, quartz powder, fly ash, nano silica, aggregate, water, fiber, superplasticizer, temperature, and age. The output is the compressive strength of UHPC. Table 2 gives the dataset description.

### 2.8. Performance Evaluation

In this study, the coefficient of determination (R^2^) is used for performance evaluation. R^2^ is a performance criterion used to compare the closeness between predicted values and experimental data. Calculation of this metric is given by Equation (1). In Equation (1), *y_i_* and ${\tilde{y}}_{i}$ are the true and predicted values of a variable. Assuming these values are stored in vector format, *n* denotes the length of these vectors.

(1) $$R^{2}={\left(\frac{n\sum_{i=1}^{n}y_{i}{\tilde{y}}_{i}-\sum_{i=1}^{n}y_{i}\sum_{i=1}^{n}{\tilde{y}}_{i}}{\sqrt{n\sum_{i=1}^{n}y_{i}^{2}-{\left(\sum_{i=1}^{n}y_{i}\right)}^{2}}\sqrt{n\sum_{i=1}^{n}{{\tilde{y}}_{i}}^{2}-{\left(\sum_{i=1}^{n}{\tilde{y}}_{i}\right)}^{2}}}\right)}^{2}$$

## 3. Results

**Figure 7:**
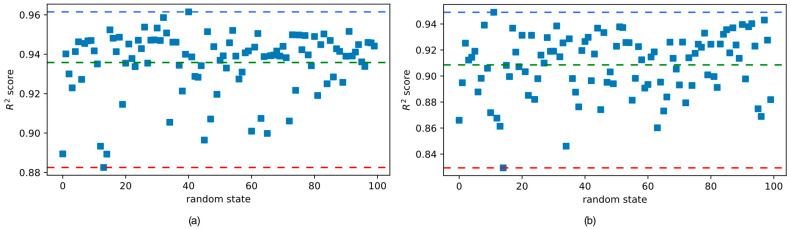
MLP performances for different random states on the (**a**) training set and (**b**) test set.

The dataset consisting of 890 data points has been randomly split into training and test sets in a 70% to 30% ratio. The predictive models have been trained on the training set whereas the test set has been used for performance evaluation. In this procedure, the model performance largely depends on the selection of the training and test samples. Therefore, in order to obtain a better understanding of the model performance, the dataset has been randomly split 100 times using the random state variable. Figure 7a,b show the model performance for each one of these random states on the training and test sets, respectively, for the multilayer perceptron (MLP) model. Figure 7b shows that the average R^2^ score of MLP for all 100 random states is 0.909, which is shown with a dashed green line. The best R^2^ score of 0.949 and the worst R^2^ score of 0.829 for the test set are shown with blue and red dashed lines, respectively.

**Figure 8:**
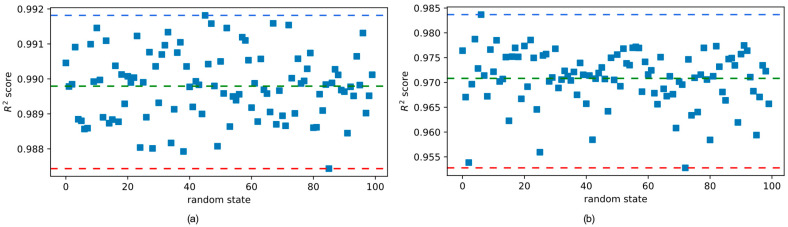
Stacking regressor performances for different random states on the (**a**) training set and (**b**) test set.

Similarly, Figure 7a shows that the average R^2^ score of MLP for all 100 random states is 0.936 which is shown with a dashed green line. The best R^2^ score of 0.962 and the worst R^2^ score of 0.883 are shown with blue and red dashed lines, respectively, for the training set. The variation in the model performance with the random state of the training set/test set split has been visualized in Figure 8 for the stacking regressor. The stacking regressor combines the outputs of the XGBoost, CatBoost, LightGBM, and Extra Trees regressors using a random forest regressor as the final estimator. Figure 8a shows that the R^2^ score of the stacking regressor fluctuates between the maximum value of 0.992 shown with a blue dashed line and the minimum value of 0.987 shown with a red dashed line. The average value of the R^2^ score on the training set is 0.990, which is shown with a green dashed line in Figure 8a. The performance of the stacking regressor on the test set is shown in Figure 8b. The best performance on the test set was an R^2^ score of 0.984, whereas the lowest R^2^ score was 0.953. The average R^2^ score on the test set was 0.971.

**Figure 9:**
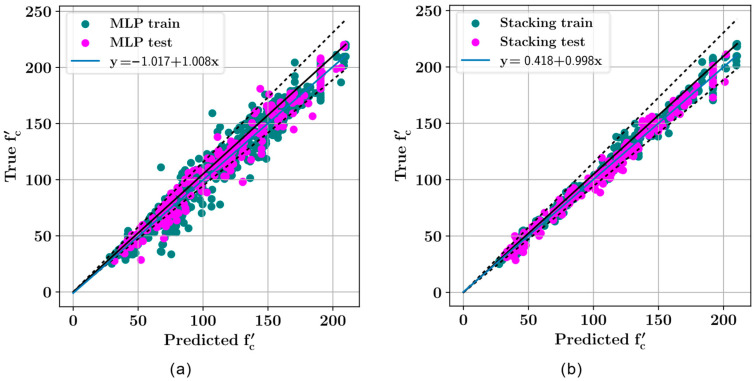
Regression plots for (**a**) MLP and (**b**) stacking regressor.

From Figure 7 and Figure 8, it can be observed that, while the selection of the random state has a considerable effect on the model performance, the stacking regressor was overall a better predictor for the compressive strength of ultra-high-performance concrete. The performances of both predictive models have been visualized in Figure 9 on both the training and the test sets. In Figure 9, the data points of the training and test sets are plotted in different colors. The predicted values of the compressive strength are plotted along the horizontal axis whereas the true values are plotted along the vertical axis. A straight diagonal line shows the perfect agreement between the predicted and true values, whereas ±10 deviation from the perfect agreement are plotted with dashed lines.

In order to quantify the impacts of different input features on the model predictions, Shapley additive explanations (SHAP) analysis has been carried out. SHAP is a methodology based on game theory which explains the output of predictive models. It is based on the concept of Shapley values from cooperative game theory, which are used to fairly allocate the contribution of each player to the overall outcome. In the context of machine learning, the input features represent the players. The formula for the calculation of the Shapley values $\phi_{i}$ can be expressed as Equation (2) [46].

(2) $$\phi_{i}=\sum_{S\subseteq F\backslash \{i\}}\frac{\left|S\right|!\left(\left|F\right|-\left|S\right|-1\right)!}{\left|F\right|!}\left[f_{S\cup \left\{i\right\}}\left(x_{S\cup \left\{i\right\}}\right)-f_{S}\left(x_{S}\right)\right]$$

**Figure 10:**
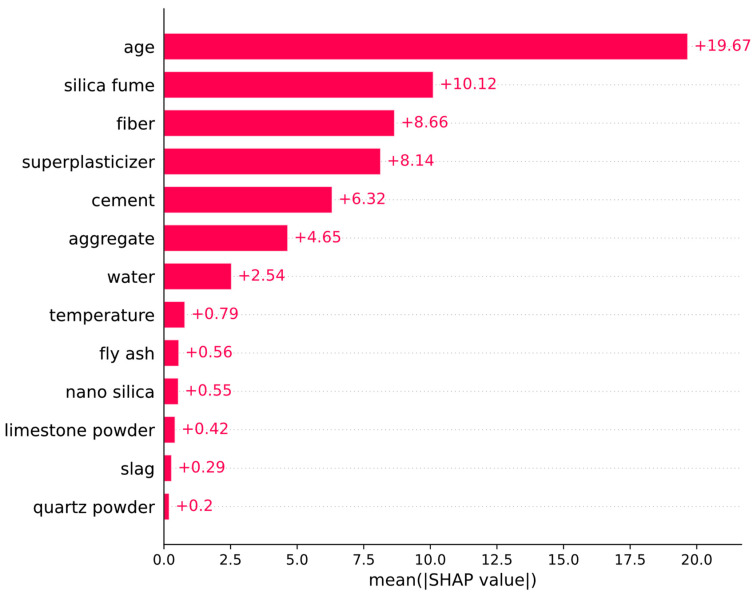
SHAP feature importances.

**Figure 11:**
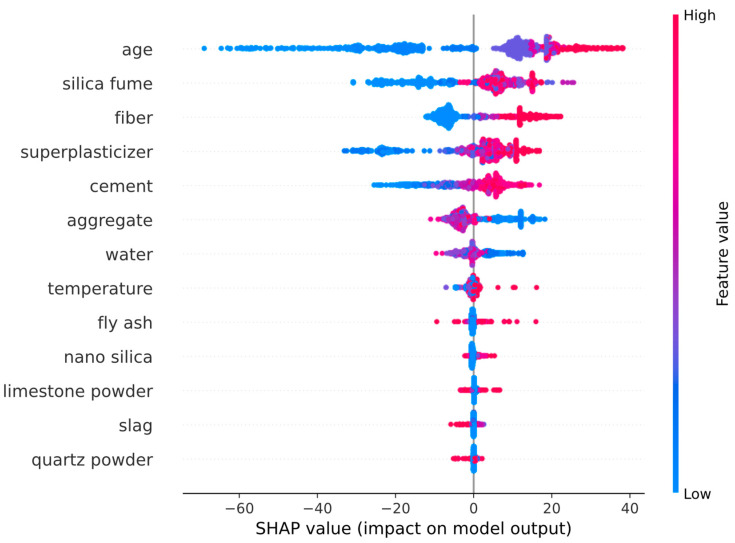
SHAP summary plot.

**Figure 12:**
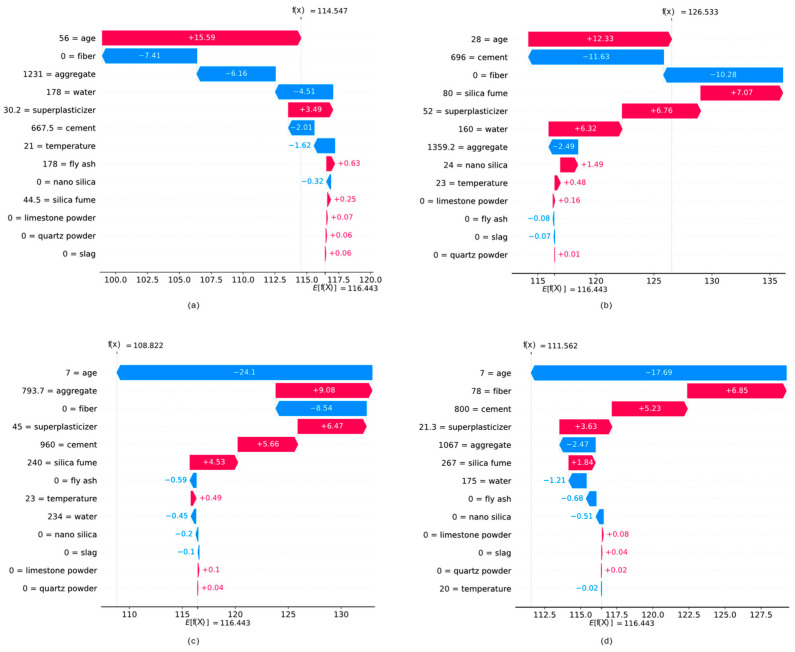
Waterfall plots for local interpretation in data points with index (**a**) i = 200, (**b**) i = 400, (**c**) i = 600, and (**d**) i = 800.

**Figure 13:**
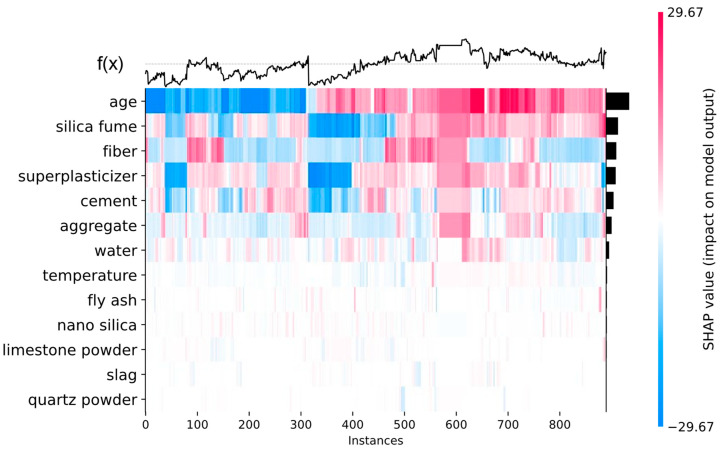
Heatmap plot for global interpretation.

In Equation (2), F is the set of all input features; S is a subset of F, which does not contain the feature with index I; **x** is a vector of feature values; and f is a function that represents the predictive model. The SHAP methodology explains the feature contributions to a model prediction on both local and global levels. The global interpretation of the feature contributions can be visualized through feature importance plots, summary plots, and heatmap plots, as shown in Figure 10, Figure 11 and Figure 12, respectively. The global interpretation plots provide an overview of the feature impacts across the entire dataset, whereas local interpretation plots provide information about the feature impacts in specific data points. The waterfall plots shown in Figure 13 are an example of local interpretation plots.

The feature importance plot in Figure 10 shows that the age of concrete and the amounts of silica fume, fiber, and superplasticizer are the most impactful input features. In Figure 10, the length of a horizontal bar represents the mean absolute SHAP value of an input feature across the entire dataset. It can be observed that the amounts of quartz powder, slag, limestone powder, and nano silica have the least impact on the model prediction.

A similar visualization technique for the feature impacts on the model output can be seen in the SHAP summary plot provided in Figure 11. In Figure 11, every dot represents one of the data points, and the color of a dot represents the value of a feature in a particular data point. High-feature values are shown in tones of red, while low-feature values are shown in tones of blue. Positive SHAP values indicate an increasing effect of a feature on the model output, while negative SHAP values indicate a decreasing effect on the model output. The distance of a data point from the vertical zero line of the SHAP values indicates the magnitude of the impact a feature has on the model prediction. According to Figure 11, increasing the age and the amounts of silica fume, fiber, superplasticizer, and cement have an increasing effect on the compressive strength. On the other hand, increasing the amounts of aggregate and water have a decreasing effect on the compressive strength. An overview of Figure 10 and Figure 11 shows that the age of the concrete and the amounts of silica fume, fiber, superplasticizer, cement, aggregate, and water have significantly more impact than the remaining input features.

The heatmap plot in Figure 12 shows an alternative representation of feature impacts. In Figure 12, every data point is represented with a vertical line along the horizontal axis. As opposed to the summary plot in Figure 11, in the heatmap plot, the colors of the data points indicate their SHAP values. For each data point, the prediction of the ML model is shown as the function f(x) on top of the graph. It can be observed that in those data instances where the most impactful features have positive SHAP values, the model predicts above-average values, as shown with f(x).

The waterfall plots in Figure 13 show the contribution of each input feature to the model prediction for four different data points, with indices 200, 400, 600, and 800. It can be seen that in data points 200 and 400, the age feature has relatively high values (56 days and 28 days), and it also has an increasing effect on the model prediction. On the other hand, in data points 600 and 800, the age feature has relatively low values (7 days), and it has a decreasing effect on the model prediction. As another example of local interpretation, we can observe that on those data instances with fiber, the inclusion of this feature in the mix has an increasing effect on the model prediction.

## 4. Discussion

The performances of a stacking regressor that combines the outputs of four different ensemble learning models has been compared to the multilayer perceptron neural network model. It was shown that the stacking regressor model can perform about 7% better than the MLP model in terms of the coefficient of determination (R^2^ score). It is known that machine-learning models can perform differently as the dataset changes. In order to obtain a better understanding of the model performances, the dataset was randomly split into a training and a test set 100 times, and the average performances were calculated. The performance of the stacking regressor on the test set was observed to fluctuate up to 3.3%, whereas the performance of the MLP regressor was observed to fluctuate up to 14.5% on the test set. Using the SHAP technique, the effects of different input features on the model output have also been investigated using summary plot, feature importance plot, heatmap plot, and waterfall plot approaches on a global and local level. The global feature importance plots showed that the age of concrete and the amounts of silica fume, fiber, superplasticizer, cement, aggregate, and water have significantly greater impact on the model predictions compared to the remaining features. The results obtained from SHAP analysis can also be helpful to determine the optimum mix ratio to achieve the desired compressive strength. Knowing the importance of the inputs will reduce the need for experimentation and save time and materials.

In previous research, Shen et al. [18] used XGBoost, AdaBoost, and Bagging to predict the compressive strength of UHPC. Among these models, the authors achieved the highest success with XGBoost, with an R^2^ of 0.90. When compared with [18], our results demonstrate a significant improvement in prediction performance.

## 5. Conclusions

Data-driven machine-learning models present an efficient means of structural performance prediction. Data-driven methods of machine learning can be used in diverse areas of structural engineering, such as biomechanics, aerospace structures, and civil engineering structures. The current study demonstrated the applicability of state-of-the-art ensemble learning and neural network prediction techniques to the compressive strength prediction of ultra-high-performance concrete. In this study, a stacking regressor ensemble model was developed using a dataset of 890 samples of compressive strength measurements. The performance of this model was compared to a multilayer perceptron neural network developed on the same dataset. In order to eliminate the effect of random dataset splits on the model performance, the dataset was split into 100 different training set/test set pairs. On average, the stacking regressor that combines the outputs of XGBoost, CatBoost, LightGBM, and Extra Trees regressors using a random forest regressor was found to perform 6.8% better than the MLP regressor on the test set, with an R^2^ score of 0.971. It should be noted that, although these results are promising, their applicability is limited within the bounds of the dataset on which the predictive models were trained. Future studies in this area can include the training of more advanced predictive models on larger datasets. The availability of high-quality datasets can introduce machine-learning techniques as a viable supplementary tool to classical methods of structural analysis.

## Figures and Tables

**Table 1:** Mechanical properties of UHPC [3].

Mechanical Properties	UHPC
Compressive strength (MPa)	200–800
Elasticity modulus (GPa)	60–75
Flexural strength (MPa)	50–140
Fracture energy (J/m^2^)	1200–40,000

**Table 2:** Dataset description.

Variable	Description
Cement	A binding material
Slag	A by-product of the smelting of metals or ores containing metals, which is a complex of oxides and silicates lighter than the metal and deposited on the surface due to density difference [45].
Silica fume	Micro-sized material that can be used in concrete as mineral admixture and pozzolanic admixture.
Limestone powder	A fine powder obtained by pulverizing clay and other materials by heat treatment in a furnace at high temperatures.
Quartz powder	A micronized powder made of natural quartz.
Fly ash	An artificial pozzolan used as a mineral admixture in concrete.
Nano silica	Material consisting of high purity amorphous silica powder.
Aggregate	Materials such as sand, gravel, and crushed stone used in concrete production.
Water	The higher the water/cement ratio, the lower the concrete strength.
Fiber	Improves the properties of concrete.
Superplasticizer	Reduces the water/cement ratio of high-performance concrete to provide very high compressive strength.
Temperature	Temperature affects the properties of concrete.
Age	Time until the concrete reaches sufficient strength.

## Data Availability

Data are available on request to authors.

## References

[1] European Aluminium Association (2006). Aluminium in Cars. 2006: Sustainability of the European Aluminium Industry.

[2] Barr A.E., Lohmann Siegel K., Danoff J.V., McGarvey C.L., Tomasko A., Sable I., Stanhope S.J. (1992). Biomechanical comparison of the energy-storing capabilities of SACH and Carbon Copy II prosthetic feet during the stance phase of gait in a person with below-knee amputation. Phys. Ther..

[3] Richard P., Cheyrezy M. (1995). Composition of reactive powder concretes. Cem. Concr. Res..

[4] Liu J., Shi C., Wu Z. (2019). Hardening, microstructure, and shrinkage development of UHPC: A review. J. Asian Concr. Fed..

[5] Nilsson L. (2018). Development of UHPC Concrete Using Mostly Locally Available Raw Materials. Master’s Thesis.

[6] Holbrook G. Hämtat från ASCE Pittsburgh Section. https://www.asce-pgh.org/.

[7] Nematollahi B., Saifulnaz M.R., Jaafar S., Voo Y.L. (2012). A review on ultra high performance ‘ductile’ concrete (UHPdC) technology. Int. J. Civ. Struct. Eng..

[8] Matte V., Richet C., Moranville M., Torrenti J.M. (1998). Characterization of reactive powder concrete as a candidate for the storage of nuclear wastes. Symposium on High-Performance and Reactive Powder Concretes.

[9] Kocataşkın F. (1991). Composition of High Strength Concrete.

[10] Huiskes R., van Rietbergen B. (2005). Biomechanics of bone. Basic Orthop. Biomech. Mechano-Biol..

[11] Turner C.H., Burr D.B. (1993). Basic biomechanical measurements of bone: A tutorial. Bone.

[12] Rincón-Kohli L., Zysset P.K. (2009). Multi-axial mechanical properties of human trabecular bone. Biomech. Model. Mechanobiol..

[13] Yuvaraj P., Murthy A.R., Iyer N.R., Samui P., Sekar S.K. (2014). Prediction of fracture characteristics of high strength and ultra high strength concrete beams based on relevance vector machine. Int. J. Damage Mech..

[14] Marani A., Jamali A., Nehdi M.L. (2020). Predicting ultra-high-performance concrete compressive strength using tabular generative adversarial networks. Materials.

[15] Solhmirzaei R., Salehi H., Kodur V., Naser M.Z. (2020). Machine learning framework for predicting failure mode and shear capacity of ultra high performance concrete beams. Eng. Struct..

[16] Jiang C.S., Liang G.Q. (2021). Modeling shear strength of medium-to ultra-high-strength concrete beams with stirrups using SVR and genetic algorithm. Soft Comput..

[17] Kumar A., Arora H.C., Kapoor N.R., Mohammed M.A., Kumar K., Majumdar A., Thinnukool O. (2022). Compressive Strength Prediction of Lightweight Concrete: Machine Learning Models. Sustainability.

[18] Shen Z., Deifalla A.F., Kamiński P., Dyczko A. (2022). Compressive strength evaluation of ultra-high-strength concrete by machine learning. Materials.

[19] Liu B. (2023). Estimating the ultra-high-performance concrete compressive strength with a machine learning model via meta-heuristic algorithms. Multiscale Multidiscip. Model. Exp. Des..

[20] Hiew S.Y., Teoh K.B., Raman S.N., Kong D., Hafezolghorani M. (2023). Prediction of ultimate conditions and stress–strain behaviour of steel-confined ultra-high-performance concrete using sequential deep feed-forward neural network modelling strategy. Eng. Struct..

[21] Zhu H., Wu X., Luo Y., Jia Y., Wang C., Fang Z., Zhuang X., Zhou S. (2023). Prediction of early compressive strength of ultrahigh-performance concrete using machine learning methods. Int. J. Comput. Methods.

[22] Nguyen M.H., Nguyen T.A., Ly H.B. (2023). Ensemble XGBoost schemes for improved compressive strength prediction of UHPC. Structures.

[23] Gong N., Zhang N. (2023). Predict the compressive strength of ultra high-performance concrete by a hybrid method of machine learning. J. Eng. Appl. Sci..

[24] Ye M., Li L., Yoo D.Y., Li H., Zhou C., Shao X. (2023). Prediction of shear strength in UHPC beams using machine learning-based models and SHAP interpretation. Constr. Build. Mater..

[25] Zhang Y., An S., Liu H. (2024). Employing the optimization algorithms with machine learning framework to estimate the compressive strength of ultra-high-performance concrete (UHPC). Multiscale Multidiscip. Model. Exp. Des..

[26] Nguyen N.H., Abellán-García J., Lee S., Vo T.P. (2024). From machine learning to semi-empirical formulas for estimating compressive strength of Ultra-High Performance Concrete. Expert Syst. Appl..

[27] Li Y., Yang X., Ren C., Wang L., Ning X. (2024). Predicting the Compressive Strength of Ultra-High-Performance Concrete Based on Machine Learning Optimized by Meta-Heuristic Algorithm. Buildings.

[28] Wakjira T.G., Alam M.S. (2024). Performance-based seismic design of Ultra-High-Performance Concrete (UHPC) bridge columns with design example–Powered by explainable machine learning model. Eng. Struct..

[29] Zhu P., Cao W., Zhang L., Zhou Y., Wu Y., Ma Z.J. (2024). Interpretable Machine Learning Models for Prediction of UHPC Creep Behavior. Buildings.

[30] Das P., Kashem A. (2024). Hybrid machine learning approach to prediction of the compressive and flexural strengths of UHPC and parametric analysis with shapley additive explanations. Case Stud. Constr. Mater..

[31] McCulloch W.S., Pitts W. (1943). A logical calculus of the ideas immanent in nervous activity. Bull. Math. Biophys..

[32] Géron A. (2022). Hands-on Machine Learning with Scikit-Learn, Keras, and TensorFlow.

[33] Deng L., Yu D. (2014). Deep learning: Methods and applications. Found. Trends® Signal Process..

[34] Car Z., Baressi Šegota S., Anđelić N., Lorencin I., Mrzljak V. (2020). Modeling the spread of COVID-19 infection using a multilayer perceptron. Comput. Math. Methods Med..

[35] Heidari A.A., Faris H., Aljarah I., Mirjalili S. (2019). An efficient hybrid multilayer perceptron neural network with grasshopper optimization. Soft Comput..

[36] Wolpert D.H. (1992). Stacked generalization. Neural Netw..

[37] Witten I.H., Frank E., Hall M.A., Pal C.J., Data M. (2005). Practical machine learning tools and techniques. Data Mining.

[38] Kumar A., Mayank J. (2020). Ensemble Learning for AI Developers.

[39] Chen T., Guestrin C. Xgboost: A scalable tree boosting system. Proceedings of the 22nd Acm Sigkdd International Conference on Knowledge Discovery and Data Mining.

[40] Jabeur S.B., Mefteh-Wali S., Viviani J.L. (2024). Forecasting gold price with the XGBoost algorithm and SHAP interaction values. Ann. Oper. Res..

[41] Zhou F., Pan H., Gao Z., Huang X., Qian G., Zhu Y., Xiao F. (2021). Fire prediction based on catboost algorithm. Math. Probl. Eng..

[42] Ke G., Meng Q., Finley T., Wang T., Chen W., Ma W., Ye Q., Liu T.Y. (2017). Lightgbm: A highly efficient gradient boosting decision tree. Adv. Neural Inf. Process. Syst..

[43] Geurts P., Ernst D., Wehenkel L. (2006). Extremely randomized trees. Mach. Learn..

[44] Abul K., Rezaul K., Chandra M.S., Pobithra D. (2023). *Ultra-High-Performance Concrete (UHPC)*, version 1; Mendeley Data.

[45] Ünal A. (2017). From Waste to Product Iron-Steel’s Slag. Master’s Thesis.

[46] Lundberg S.M., Lee S.-I. A unified approach to interpreting model predictions. Proceedings of the 31st Conference on Neural Information Processing Systems (NIPS 2017).

